# Seasonal changes in neophobia and its consistency in rooks: the effect of novelty type and dominance position

**DOI:** 10.1016/j.anbehav.2016.08.010

**Published:** 2016-11

**Authors:** Alison L. Greggor, Jolle W. Jolles, Alex Thornton, Nicola S. Clayton

**Affiliations:** a Department of Psychology, University of Cambridge, Cambridge, U.K.; b Department of Collective Behaviour, Max Planck Institute for Ornithology, Konstanz, Germany; c Centre for Ecology and Conservation, University of Exeter, Penryn, U.K.

**Keywords:** dominance, individual consistency, neophobia, predatory wariness, seasonal change

## Abstract

Neophobia, or the fear of novelty, may offer benefits to animals by limiting their exposure to unknown danger, but can also impose costs by preventing the exploration of potential resources. The costs and benefits of neophobia may vary throughout the year if predation pressure, resource distribution or conspecific competition changes seasonally. Despite such variation, neophobia levels are often assumed to be temporally and individually stable. Whether or not neophobia expression changes seasonally and fluctuates equally for all individuals is crucial to understanding the drivers, consequences and plasticity of novelty avoidance. We investigated seasonal differences and individual consistency in the motivation and novelty responses of a captive group of rooks, *Corvus frugilegus*, a seasonally breeding, colonial species of corvid that is known for being neophobic. We tested the group around novel objects and novel people to determine whether responses generalized across novelty types, and considered whether differences in dominance could influence the social risk of approaching unknown stimuli. We found that the group's level of object neophobia was stable year-round, but individuals were not consistent between seasons, despite being consistent within seasons. In contrast, the group's avoidance of novel people decreased during the breeding season, and individuals were consistent year-round. Additionally, although subordinate birds were more likely to challenge dominants during the breeding season, this social risk taking did not translate to greater novelty approach. Since seasonal variation and individual consistency varied differently towards each novelty type, responses towards novel objects and people seem to be governed by different mechanisms. Such a degree of fluctuation has consequences for other individually consistent behaviours often measured within the nonhuman personality literature.

## Highlights

• Seasonal variation and consistency in neophobia was measured in a rook social group.
• Object neophobia did not vary seasonally but fear of novel people did.
• Dominance did not predict neophobia.
• Individuals expressed contrasting reaction norms for different types of avoidance.
• Seasonal variation and inconsistency suggest neophobia is not always a stable trait.

When animals express neophobia, or the fear of novelty, they show an aversion to an unknown risk (Greenberg & Mettke-Hofmann, 2001). Since species have been shaped over evolutionary time to avoid unknown risks, neophobia is often thought to drive species level traits such as niche breadth, home range size or dietary generalism (Greenberg, 1989, Greenberg, 1990, Greenberg, 1992, Greenberg and Mettke-Hofmann, 2001). For example, high levels of neophobia may be favoured by selection in habitats where increased wariness is beneficial for survival and reproduction, for example in predator-rich environments (Ferrari, McCormick, Meekan, & Chivers, 2015). However, elevated neophobia may also carry potential costs if increased fear inhibits innovation (Benson-Amram and Holekamp, 2012, Greenberg, 2003), or limits defences, for instance, against nest predators (Vrublevska et al., 2015). These costs and benefits of risk taking are likely to vary over time and contexts in a way that could alter the expression of neophobia. For example, it could be beneficial to adjust neophobia levels when environmental opportunities or dangers change, such as food availability or predation pressure (e.g. Brown, Ferrari, Elvidge, Ramnarine, & Chivers, 2013). Therefore, animals may have evolved species-typical patterns of plasticity in neophobia if environments vary in predictable ways.

Every year environments undergo predictable seasonal cycles that trigger changes in animals' metabolism and thermoregulatory processes (Thomas, Bieber, Arnold, & Millesi, 2012). Therefore, just as seasonal change impacts behaviour related to physiological processes, neophobia levels may also change in response to the changing risks and rewards of the time of year. The extent to which species mediate their neophobia seasonally is unclear, and the handful of studies conducted on birds to date have generated conflicting and inconsistent findings (Apfelbeck and Raess, 2008, Mettke-Hofmann, 2007, Mettke-Hofmann, 2000, Shephard et al., 2014). Moreover, it is unknown whether or not all individuals respond similarly to seasonal influences.

Individuals are commonly assumed to vary consistently in their neophobia (e.g. Bebus, Small, Jones, Elderbrock, & Schoech, 2016). In fact, neophobia is often used as a marker of nonhuman personality or temperament, because it is considered a stable response to challenges or risks across times or situations (Dall, Houston, & McNamara, 2004). However, it is unclear whether all individuals similarly mediate their neophobic behaviours under changing conditions. Such individual variation begs the question of why certain behaviours remain rigid and why others show variable plasticity (Carter, Goldizen, & Heinsohn, 2012).

Several proximate and ultimate explanations for neophobic behaviour suggest species' neophobia levels should vary seasonally, and that not all individuals may be consistent in these changes. First, changes in motivation and hormone levels throughout the year could have a powerful influence on neophobia and other types of risk taking. For example, many bird species undergo physiological and behavioural changes during the breeding season (Pdulka, Rohrbaugh, & Bonney, 2004), altering hunger and activity levels, which could contribute to changes in neophobic behaviours. Levels of stress hormones, such as corticosterone, thought to influence neophobic responses, vary by season (Romero, 2002), and often lack consistency within individuals beyond seasons (Ouyang, Hau, & Bonier, 2011). In line with these patterns, over short periods of time, neophobia measures have been shown to be highly consistent (e.g. Jolles, Ostojić, & Clayton, 2013, although see Miller, Bugnyar, Pölzl, & Schwab, 2015), while over longer timeframes such as years, they can lack such consistency (e.g. Kluen & Brommer, 2013).

Second, seasonal changes to animals' social systems could influence the risks and rewards of approaching novelty. For example, the presence of dominant individuals can alter the costs or benefits of neophobia if approaching novelty allows subordinates to circumvent competition for favoured resources, but this can depend on the species in question. In some corvid social systems, such as those of carrion crows, *Corvus corone*, dominants are more likely to take risks by approaching novelty, and subordinates benefit, at least in family groups (Chiarati, Canestrari, Vera, & Baglione, 2012). However, in other species, such as common ravens, *Corvus corax*, subordinates are less neophobic, at least around novel food, potentially approaching novelty to avoid competition with dominants (Heinrich, Marzluff, & Adams, 1995). If seasonal changes in social structure and hormone levels increase the frequency of contact and aggression between subordinates and dominants, then the risks and rewards for approaching novelty might also vary, but would do so differently depending on individuals' dominance rank. Additionally, the presence of conspecifics can influence levels of novelty approach (Miller et al., 2015), and the extent to which conspecific social cues influence behaviour can vary seasonally (e.g. Greggor, McIvor, Clayton, & Thornton, 2016). Therefore, efforts to determine the factors that influence neophobia must consider the dominance of individuals, and the social environment they occupy when assessing risk taking. By measuring neophobia within social settings that would be common in the wild (Dall & Griffith, 2014), tests are more likely to capture natural interactions between dominance, neophobia and seasonal changes to the social system.

Finally, not all types of novel stimuli elicit the same reactions, and different types of novelty may be more threatening at certain times of year. Individual measures of neophobia towards different types of novelty, such as objects and locations, do not always correlate (e.g. Boogert et al., 2006, Fox et al., 2009), and neophobia is not always predictive of wariness towards other threatening stimuli such as predators (e.g. Carter, Marshall, Heinsohn, & Cowlishaw, 2012). Similar to what has been proposed for other behaviours considered to be stable across time and/or contexts (Dall & Griffith, 2014), understanding the mechanisms behind neophobic behaviour requires examining it when contextual changes occur that may influence its expression. Several underlying mechanisms can contribute to the expression of neophobic behaviour, such as novelty categorization and physiological fear responses (Greggor, Thornton, & Clayton, 2015). Individual fluctuation in these mechanisms could help explain the existence and maintenance of individually varying behavioural reaction norms (e.g. Dingemanse, Kazem, Reale, & Wright, 2010). However, without an understanding of how neophobia naturally varies throughout the year, it is difficult to assess to what extent individuals might vary in their level and stability of neophobia.

We measured the risk-taking behaviour of a social group of captive rooks, *Corvus frugilegus*, towards novel objects and novel people to measure the temporal effects and individual stability of neophobia. Tests and their control conditions were run over a full year within a social group to gauge the potential effect of social rank on neophobia over time. While novel object tests are the most common measure of neophobia (Greggor et al., 2015), examining reactions to novel people allowed us to verify whether seasonal change influences novelty responses per se, or influences more ecologically relevant fear behaviours such as predatory wariness. Rooks are an excellent model species to test these dynamics because they experience seasonal changes in behaviour while breeding, are known to be very neophobic (Greggor, Clayton et al., 2016, Jolles et al., 2013), and are likely to be able to discriminate between human faces, as other corvids do (Davidson et al., 2015, Lee et al., 2011, Marzluff et al., 2010). Moreover, since we tested a group from which data had previously been collected on neophobia and dominance in the context of social feeding tactics (Jolles et al., 2013), we were also able to compare selected behaviours across a 4-year period.

Our experimental set-up led to a set of four predictions. We predicted that (1) the rooks would be more likely to approach novel objects and people during the breeding season, because hunger and feeding rates increase at that time (Feare, Dunnet, & Patterson, 1974), which can increase risk taking (Damsgard & Dill, 1998). Additionally, we predicted (2) that subordinates would demonstrate lower neophobia to avoid competition with dominants (i.e. a similar situation to ravens, Heinrich et al., 1995), but expected this effect to depend on the season, as subordinates might be more willing to risk competing with dominant individuals during the breeding season. We also predicted that (3) individual consistency across seasons would differ depending on the type of novelty. Despite both stimuli being novel, reactions towards novel people may also elicit reactions of predatory wariness, which does not always correlate with neophobia (Carter, Marshall, et al., 2012), and could be subject to different seasonal pressures. Finally, we predicted that (4) individuals would not be consistent in their approach behaviour across the different types of novelty because avoidance towards objects versus people could involve different cognitive mechanisms and ecological biases whose response strength may vary independently between individuals.

## Methods

### Subjects and Housing

The group of adult rooks was housed in an outdoor aviary at the University of Cambridge's Sub-Department of Animal Behaviour, Madingley, U.K. where they experienced ambient light and temperature fluctuations throughout the year, and viewed woodlands and natural predators from the enclosure. The birds were collected as chicks from two Cambridgeshire colonies in 2003 and were hand-raised. The birds were given sticks for nest building during the breeding season, and they formed pairs and laid eggs. During this time, they were highly active in maintaining and defending their nests, since a fixed amount of high-quality sticks was available to the group. Eggs were pricked upon discovery (in accordance with Home Office animal welfare regulations) so that no birds were actively rearing young. As part of regular health checks, the rooks had to be caught with nets, which was a stressful experience that would be likely to mirror levels of fear experienced during predation events. The group consisted of 19 birds when initial data on dominance and object neophobia were collected in the 2010 breeding season by Jolles et al. (2013). After this, three changes in the group occurred: two birds died in 2013 (group *N* = 17); and during 2014, the data collection period for the main seasonal comparison in this study, one bird died in the summer (group *N* = 16); and two new birds were added in the autumn (group *N* = 18) from a similar aviary on the premises.

The aviary (8 × 20 m and 3 m high) was constructed of wood and mesh with a gravel floor, and had several perches and platforms at different heights, and three feeding tables 1.1 m off the ground. Birds were given colour leg rings to enable individual identification. Birds had ad libitum access to water and food except during the experimental procedures when the group was deprived for up to 4 h.

### Ethical Note

The chicks were collected under Natural England permit 20030108. The experiments were conducted in accordance with the University of Cambridge's animal welfare guidelines as nonregulated procedures under the U.K. Home Office project licence PPL 80/1975 and adhere to the standards set forth by the ASAB/ABS (2012) Guidelines for the Treatment of Animals in Behavioural Research and Teaching.

### Experimental Procedure

Experimental trials took place at one of six locations within the aviary: three on the ground and three on a feeding table. The open aviary with numerous perches provided all group members with views of the test locations without them having to approach the test stimuli. All locations were used on each test day, and their order was randomly determined beforehand. Since birds preferred to occupy different parts of the aviary, testing in multiple locations gave the greatest number of birds a chance to participate. The group was deprived of food for at least 90 min prior to the start of that day's experiments. Each test day was randomly assigned to six novelty response trials or six motivational control trials (detailed below). Although this type of control is sometimes used to validate neophobia tests (e.g. Cole & Quinn, 2014), it also serves as a measure of motivation, and therefore helps determine whether changes to motivation could explain any seasonal changes in neophobia that might occur. Additionally, since multiple birds could feed simultaneously and dominant birds could defend the food in both novelty and motivational control conditions, comparisons of behaviour between the two allowed us to determine how novel stimuli influenced the acquisition of food when these social dynamics are present.

All experiments were filmed with a Panasonic HDC-SD90 camcorder from outside the aviary, and later video coded. Birds were deemed to approach if they came within 1 m of the food, a distance that a person, conspecific or threatening object (if it were to move) could easily travel. Each bird's approach time and food consumption were noted for each trial. The bird's approach latency was used as an indicator of avoidance, and the number of larvae consumed indicated its ability to gain access to resources within the social group context. Birds that did not approach were given a maximum time equal to the length of the trial. Intercoder reliability on approach times and food consumption was assessed by recoding a random subset of videos (31% of trials). Reliability was deemed to be very high (approach times: intraclass correlation, ICC (1) = 0.93, confidence interval, CI = 0.92–0.94; food consumption: ICC (1) = 0.92, CI = 0.91–0.93).

### Novelty Response Tests

#### Novel object test

**Figure A1:**
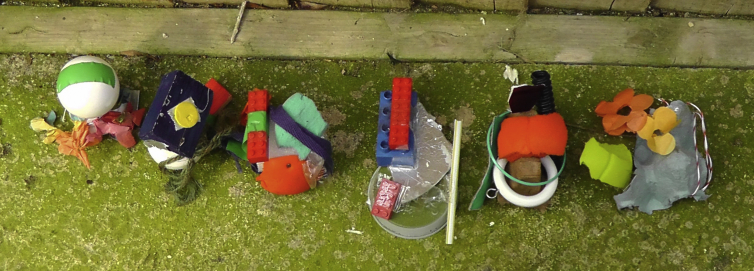
Examples of novel objects.

Object neophobia was assessed similarly to previous protocols used with the same group of birds (Jolles et al., 2013). The experimenter placed a familiar food bowl containing eight wax moth larvae (a preferred food) and the novel object in the aviary, 10 cm apart, and left the aviary. After 5 min the experimenter returned and removed the object and food bowl, even if the food had not been consumed. The experimenter then conducted another trial with a new object until all six locations had been tested. Novel objects were constructed out of bright, artificial materials that differed in colour, texture and shape, contained at least one shiny element and all of the primary colours and did not contain any parts that could look like eyes (see Fig. A1 for examples). A new novel object was used for every trial. Nonobject, motivational control trials were run in the same manner as the novel object trials, but with only food presented. Throughout 2014, a total of 42 object neophobia trials and 36 control trials were run over the course of 13 days.

#### Novel people test

Over the years, many anecdotes have accumulated indicating that the rook groups at the study site were highly wary of novel people despite having been hand-raised. New experimenters had to spend several months with the group before the majority of birds would approach and feed near them. Furthermore, there appeared to be substantial individual variation in how long birds took to take food from the hand of a new experimenter, with some birds never hand feeding despite years of interactions with an experimenter.

To quantify these tendencies and determine the extent to which they related to variation in novel object responses, six novel female experimenters each conducted a set of six feeding latency trials over the course of 2014. The birds had never seen the novel experimenters prior to the day of the trial. Each experimenter approached the aviary unaccompanied (i.e. without the presence of a known experimenter), walked to one of the six predetermined locations within the aviary, tossed five wax moth larvae 2–3 m in front of her, and remained staring at the larvae. She tossed five instead of eight larvae, the number presented in the novel object trial, because pilot trials indicated that five were easier to monitor on the gravel floor. The experimenter then waited until all five larvae were eaten and then tossed an additional five. She continued doing this for a total of 10 min. If no bird approached during that time, she left the initial five larvae and exited the aviary. She repeated the trial until all six locations had been tested. To control for differences in hunger motivation over the course of the year, control trials were run with the same protocols by a familiar female experimenter who had been working with the group consistently since the spring of 2013.

### Dominance

Dominance hierarchies were measured via ad libitum (Altmann, 1974) behavioural observations of the group. Observations were used from a previous study (Jolles et al., 2013) from summer 2010, and were collected at three additional time points: (1) breeding season 2014, (2) summer 2014 and (3) winter 2014. Behavioural observations were carried out in person from 10 m outside the aviary as well as from video recordings of morning feedings. A total of 1753 agonistic interactions were recorded throughout the year. Aggressive interactions included behaviours such as displacing, threatening or chasing other individuals (for full ethogram see Jolles et al., 2013). Aggressive interactions at nest locations were not included since birds might be expected to defend their nest even against dominant individuals.

All dominance interactions were organized in a sociometric matrix. To test for linearity we calculated Landau's index *h* and the index of linearity *h*′ using the DyaDA package (Leiva, Solanas, & Kenny, 2010). Both indices vary from 0 (complete absence of linearity) to 1 (complete linearity). The index *h*′ is based on *h* and takes into account the existence of unknown relationships. Statistical significance of *h*′ is provided by a resampling procedure using 10 000 randomizations (de Vries, 1995). When linearity of the dominance was observed, individuals' ranks were calculated such that their rank order minimized the number of inconsistencies and then minimized the total strength of inconsistencies (de Vries, 1998).

Additionally, to determine whether the levels of aggression within the group varied seasonally, the number of aggressive behaviours that occurred around the food bowl was recorded for all nonobject control trials and compared across seasons. Nonobject controls were used instead of the familiar person controls because a standardized amount of food was presented in these trials, thereby providing identical opportunities for aggression, and hence a more accurate measure of whether or not subordinates experienced a different social cost of approaching between seasons.

### Analysis

All data were analysed in R (R Core Team, 2015). In the few cases where bird identity could not reliably be determined from the video, that trial was removed from analysis (7.5% of trials had at least one uncertain bird, but this was spread evenly throughout the year).

#### Seasonal variation in behaviour

We assessed seasonal variation in behaviour by analysing birds' raw approach times and the amount of food they consumed. First, we analysed the probability that any bird would approach over time using a type of survival model, a Cox proportional hazards regression, on the raw approach time data. Survival analyses deal with data that is censored by a predetermined trial end time, which would otherwise be problematic because birds with maximum times may have approached had the trials run longer. Using survival models to analyse the approach data generated from neophobia tests is common (e.g. Bókony, Kulcsár, Tóth, & Liker, 2012). We primarily investigated the effects of experimental condition, dominance, season and any interactions between them. Although sex differences in risk taking have been found in other species (Jolles, Boogert, & van den Bos, 2015), preliminary analyses found that it covaried strongly with dominance and did not predict any of our response variables (similarly to Jolles et al., 2013). Therefore, it was not included in the final analyses. The potentially confounding covariates of trial order and aviary location were included in the model. Data were clustered around bird identity and trial to account for dependence in the data. We used the ‘survival’ package in R (Therneau, 2015).

Second, we analysed the raw data on the number of larvae consumed by each bird with generalized linear mixed models (GLMMs) with a negative binomial error structure and the same effects and covariates as the survival analysis. Bird identity and trial were included as random effects to account for repeated measures. Models were run with the glmmadmb package (Fournier et al., 2012). Any analysis that found seasonal effects was repeated without the three individuals that either left or entered the group over the year to ensure changes in group composition could not explain any seasonal variation we found. In the Results we only report this extra analysis when it produced different results.

Finally, the total counts of aggression for the control object trials were compared across seasons with a chi-square test.

#### Behavioural consistency

We assessed how consistent individuals were within seasons in their approach times in the presence of novelty and during motivational controls. The distribution of raw approach times was non-normal and highly skewed by birds that did not approach. Therefore, we assessed individuals' within-season behavioural consistency by calculating ICCs (Nakagawa & Schielzeth, 2010) using the ‘irr’ package (Gamer, Lemon, Fellows, & Singh, 2010), for each condition and stimulus type.

Additionally, we assessed how consistent individuals were between seasons and years in their dominance and their approach rankings in control and test conditions. Raw approach times were transformed into individual approach ranks for control and test conditions and compared with Spearman rank correlation tests. Approach ranks were calculated for each trial based on the order in which individuals approached the food cup. Overall ranks were determined by averaging trial ranks separately for each condition and season, accounting for the number of trials in which each bird was present. Any birds that did not approach during a given trial were given the same lowest approach rank.

Additionally, to compare birds' rankings in novel object tests and dominance status in breeding season across years, we combined our data with that from a previous study of the same group (Jolles et al., 2013). Individual rankings for this comparison were based on the number of approaches individuals made towards novel objects or the control food bowl and only birds found in both time periods of interest were used in comparisons.

Finally, we compared birds' consistency in their ranking between stimuli types (object and people tests) and between motivational controls within seasons with Spearman rank correlation tests. Only birds present in both time periods being compared were used (*N* = 17 between years, *N* = 16 between seasons). Since rank measures were used in multiple comparisons (between seasons, between years and within seasons against different stimuli types), all reported *P* values were adjusted through Holm's method (Shaffer, 1995).

## Results

### Seasonal Variation in Behaviour

#### Novel object test

**Figure A2:**
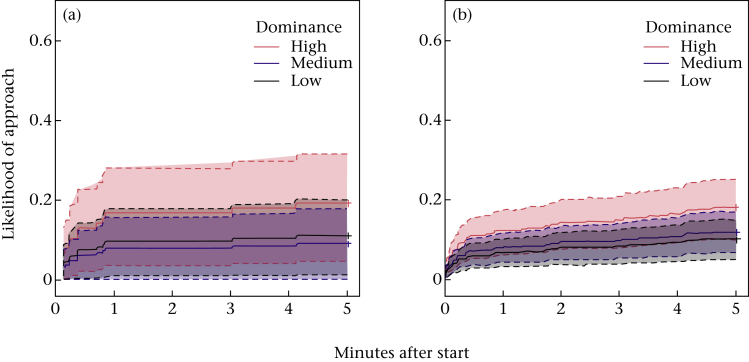
Food bowl approach by dominance in two periods of the nonbreeding season. Inverted survival curves on the restricted data set containing birds present in all seasons; likelihood that birds of different dominance rank approached the food bowl in both conditions in the (a) summer of 2014 before two individuals were added and (b) autumn of 2014 after two individuals were added. Dotted lines show confidence intervals. Dominance ranks were grouped evenly into categories of ‘high’, ‘medium’ and ‘low’ for graphical representation, but were analysed as a continuous variable.

**Figure 1:**
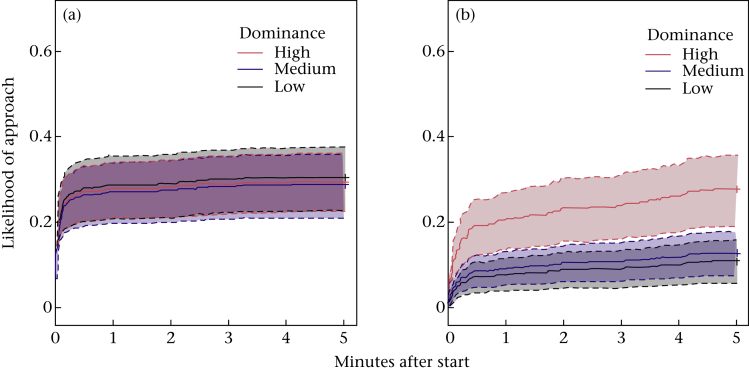
Food bowl approach. Inverted survival curves showing the likelihood that birds of different dominance ranks approached the food bowl in both object and nonobject conditions over time in the (a) breeding and (b) nonbreeding season. Dotted lines show confidence intervals. Dominance ranks were grouped evenly into categories of ‘high’, ‘medium’ and ‘low’ for graphical representation, but were analysed as a continuous variable. Only variables of interest were included in the models used to produce the graphs.

Birds were less likely to approach the food bowl when a novel object was present than when one was not, regardless of season (Cox proportional hazards model: *N* = 1303 observations, 253 events, Table 1). There was an interaction between season and dominance that held for both test and control conditions: all birds were equally likely to approach the food bowl in the breeding season, but dominant birds were more likely than subordinate birds to approach outside the breeding season (Fig. 1, Table 1). To ensure this seasonal effect was not due to changes in group composition, the same tests were conducted on the data from birds only present in all time periods. Although this interaction was no longer significant with this restricted data set (*z* = 0.06, *P* = 0.081), the smaller data set showed the same nonbreeding season trends, both before and after the addition of two birds (Fig. A2). Food consumption did not differ by season, dominance rank or any other factor. More aggressive behaviours occurred during nonobject control trials in the breeding season than outside it (31 during 18 trials versus six during 18 trials; $\chi_{1}^{2}$ = 7.97, *P* = 0.005).

#### Novel people test

**Figure 2:**
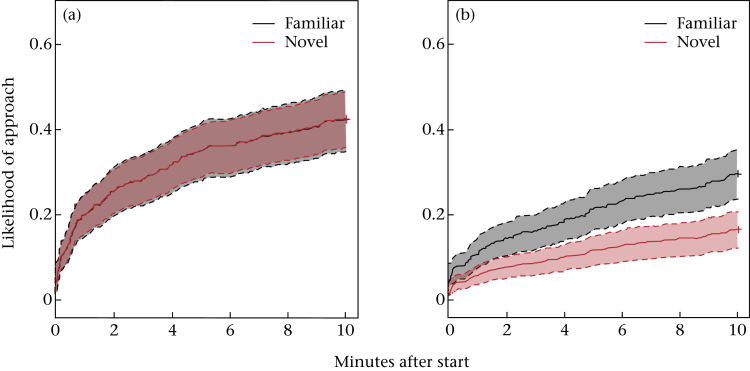
Novel and familiar people approach. Inverted survival curves showing the increasing probability that birds approached the experimenter over time, broken down by condition in the (a) breeding and (b) nonbreeding season. Dotted lines denote confidence intervals. Only variables of interest were included in models used to produce the graphs.

Birds approached novel and familiar experimenters similarly in the breeding season, but were slower to arrive around novel people in the nonbreeding season (Cox proportional hazards model, *N* = 928 observations, 299 events, Table 2, Fig. 2). Additionally, dominant birds were more likely to approach the experimenter (*z* = 2.77, *P* = 0.005), regardless of the time of year or whether the person was novel; however, the magnitude of the effect was comparatively small (see Table 2). A greater percentage of the food was eaten by dominant than subordinate birds (GLMM: *N* = 928, estimate = −0.13±0.05, *z* = −2.7, *P* = 0.007), regardless of season or condition.

### Behavioural Consistency

#### Dominance

The dominance hierarchy was linear during the 2010 breeding season (*h* = 0.57, *h*^*′*^ = 0.61, *P* < 0.001) as well as in both seasons in 2014 (breeding: *h* = 0.32, *h*^*'*^ = 0.39, *P* < 0.001; nonbreeding: *h* = 0.47, *h*^*'*^ = 0.51, *P* < 0.001). Breeding season ranks between 2010 and 2014 for birds present in both time periods were highly correlated (*r* = 0.77, CI = 0.45–0.91, *P* < 0.001). However, the dominance hierarchies between the breeding and nonbreeding seasons of 2014 were not significantly correlated (*r* = 0.42, CI = −0.10–0.76, *P* = 0.110).

#### Novel object tests

**Figure 3:**
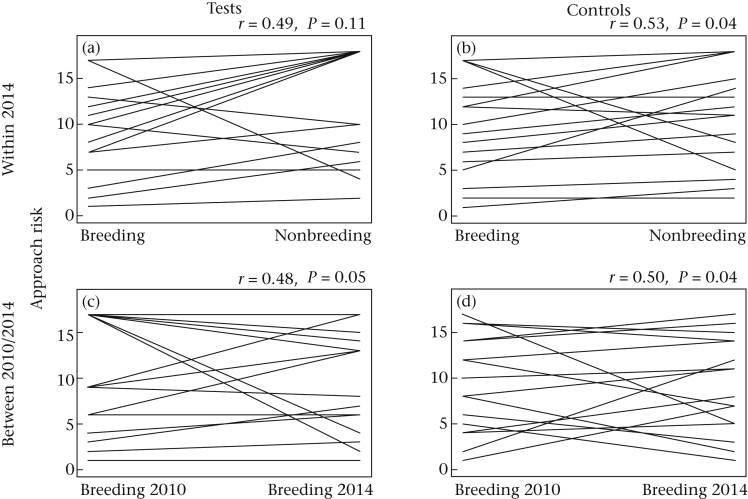
Approach rankings across time and experimental condition. Comparisons of (a) novel object test and (b) nonobject control rankings between seasons of 2014 and of (c) test and (d) control between breeding seasons of 2010 versus 2014. Correlations and *P* values are shown. Tied ranks occurred when individuals showed identical approach behaviour over the season.

Birds were consistent in their approach times during the novel object trials within the breeding season (ICC(1) = 0.28, CI = 0.16–0.49, *P* < 0.001) and within the nonbreeding season (ICC(1) = 0.15, CI = 0.06–0.34, *P* < 0.001). Birds were also consistent in their approach times during the nonobject control trials in the breeding season (ICC(1) = 0.23, CI = 0.12–0.442, *P* < 0.001), but had a very low measure of consistency during the nonbreeding season (ICC(1) = 0.06, CI = 0.01–0.19, *P* = 0.007). Across seasons in 2014, individual approach rankings were not consistent for novel object trials (*r* = 0.49, CI = −0.01–0.79, *P* = 0.105; Fig. 3a), but were consistent for nonobject controls (*r* = 0.53, CI = 0.05–0.82, *P* = 0.042; Fig. 3b). In contrast, across breeding seasons in different years (2010 versus 2014) birds' approach ranks were marginally nonsignificantly correlated during novel object trials (*r* = 0.48, CI = 0.00–0.78, *P* = 0.053; Fig. 3c), and significantly correlated during controls (*r* = 0.50, CI = 0.02–0.79, *P* = 0.042; Fig. 3d).

#### Novel people tests

Birds were consistent in how quickly they approached novel experimenters within the breeding season (ICC(1) = 0.16, CI = 0.06–0.36, *P* < 0.001), but not within the nonbreeding season (ICC(1) = 0.00, CI = 0.00–0.07, *P* = 0.533). Similarly, birds approached consistently during the breeding season in control trials with the familiar experimenter (ICC(1) = 0.24, CI = 0.11–0.46, *P* < 0.001), but showed very low levels of consistency within the nonbreeding season (ICC(1) = 0.08, CI = 0.01–0.23, *P* = 0.008). Across seasons, birds were consistent in their approach rank during novel conditions (*r* = 0.55, CI = 0.07–0.82, *P* = 0.028), but were not consistent during control conditions (*r* = 0.42, CI = −0.09–0.76, *P* = 0.105).

#### Correlations between novelty responses

**Figure 4:**
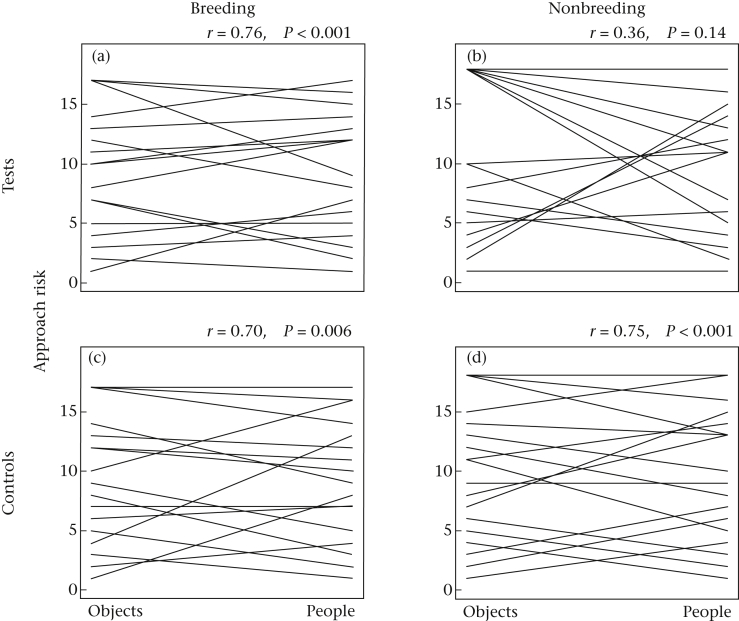
Approach rankings within seasons and across types of stimuli. Comparisons of (a) breeding and (b) nonbreeding season test rankings, and of (c) breeding and (d) nonbreeding season control ranks. Correlations and adjusted *P* values are shown. Tied ranks occurred if individuals approached or did not approach in the same frequencies.

The relationship between responses towards novel objects and novel people changed throughout the year. During the breeding season, individuals' ranks on the two novelty responses were correlated (*r* = 0.76, CI = 0.44–0.91, *P* < 0.001; Fig. 4a), but outside the breeding season they were not (*r* = 0.36, CI = −0.13–0.71, *P* = 0.144; Fig. 4b). Meanwhile, birds were consistent in their approach across both types of motivational controls (nonobject and familiar person) during both the breeding season (*r* = 0.70, CI = 0.32–0.88, *P* = 0.006; Fig. 4c) and the nonbreeding season (*r* = 0.75, CI = 0.43–0.90, *P* < 0.001; Fig. 4d).

## Discussion

Little is known about the extent to which neophobia levels vary seasonally and about whether or not all individuals respond similarly to seasonal change. We investigated seasonal changes in motivation and responses to novelty within a social group of rooks and determined the behavioural consistency of individuals across time and contexts. Both the level and consistency of individuals' risk-taking behaviour varied depending on the season, birds' dominance ranks and the type of novel stimuli used. The group was more likely to approach novel people during the breeding season, as expected, but was equally wary of novel objects in both seasons, suggesting that the breeding season may have different effects on novelty approach depending on the type of stimuli being presented. Additionally, although subordinate birds were more likely to approach a highly contested food bowl around dominants during the breeding season, they did not approach novelty more than dominants. Finally, whether or not all individuals responded similarly to these seasonal changes depended on the type of stimuli; individuals were not consistent between seasons in their novel object approach, but were consistent in their novel people approach. Overall, the season thus greatly impacted both the motivation and novelty responses of individuals, but did not always impact them equally.

The differences in motivation and neophobia that we found could stem from several seasonal changes that birds undergo, including increased hunger, altered responses to predators, changes in reactions to social cues and/or stress hormone fluctuations. For example, the increased energetic costs of breeding and maintaining a nest could cause decreases in neophobia because hunger stimulates risk taking (Damsgard & Dill, 1998). Accordingly, rooks are more likely to take risks while foraging in the wild during the breeding season (Green, 1981), and those that take risks are more likely to be in poorer body condition (Patterson, Dunnet, & Goodbody, 1988). Moreover, baseline levels of glucocorticoid stress hormones are known to be higher during the breeding season in a range of bird species (Romero, 2002) and the extent of these changes can vary depending on dominance status (Kotrschal, Hirschenhauser, & Mostl, 1998). We found evidence that subordinate birds were more motivated for food during the breeding season, because they were more willing to compete with dominants, despite the seasonally higher levels of aggression they suffered when approaching the food bowl in object neophobia tests and controls. However, unlike other studies that have found differences in neophobia by dominance status in corvids (Chiarati et al., 2012, Heinrich et al., 1995), subordinate rooks competed equally around dominants regardless of whether it was a novel object or control trial, and therefore any increases in motivation did not translate to increases in novelty approach. Subordinates' greater approach of the food bowl in the breeding season did not translate to any differences in food consumption of the highly contested larvae, potentially because social competition reduces the feeding success of subordinates more than dominants (Vahl, Lok, Van Meer, Piersma, & Weissing, 2005).

Seasonal changes in hormone levels, social cues and hunger could each influence an animal's motivation to approach novelty, but none of these clearly influenced the rooks' responses to both novel objects and novel people. Increases in the main avian glucocorticoid hormone corticosterone have been linked to reductions in boldness and increases in neophobia in previous studies (Baugh et al., 2013, Richard et al., 2008). Therefore, it could be expected that hormonal changes would contribute to increased novelty approach. However, the breeding season only promoted higher risk taking around novel people, when birds were equally quick to approach familiar and novel experimenters. In contrast, season did not have an impact on novel object approach. In closely related jackdaws, *Corvus monedula*, birds more frequently copy social cues around objects during the breeding than the nonbreeding season (Greggor, McIvor, et al., 2016). If social cues had been the driver of seasonal differences in this rook group, we would have expected novel object approach to increase in the breeding season, which it did not. Meanwhile, hunger levels should have been similar at the beginning of both types of tests because individuals were consistent when compared across both types of motivational food controls. Therefore, the contrasting relationship between seasonal change and stimuli type cannot be attributed solely to hormone levels, social cues or hunger. While these influences may alter animals' motivations across seasons, predicting what risks animals are willing to take depends on the context of risk and the type of stimulus under question.

Reactions to novel objects and novel people may involve different cognitive mechanisms, despite sharing a component of novelty (Greggor et al., 2015). The novelty of an unknown person is conflated by the fact that they are also a potential predator (Frid & Dill, 2002), and birds may be primed to take more risks around predators during the breeding season because they have nests to defend. Like wild breeding birds, the captive rooks had experience with humans as potential nest predators because their nests were unavoidably disturbed when husbandry staff pricked eggs. Therefore, the rooks might have also been primed to take more risks around novel people because they had nests to defend, and this would explain why they treated novel people similarly to familiar people during the breeding season. In contrast, unlike predation pressure which can vary reliably by season (Post & Götmark, 2006), it might be harder to predict the threat of a novel object, and therefore object neophobia may not be influenced by seasonal biases in risk taking. Moreover, if rooks of all dominance ranks share these cognitive biases, it could explain why subordinate and dominant birds did not differ in how they responded seasonally to either type of novelty.

In addition to the seasonal changes to motivation and novelty approach, we also found that individuals' levels of consistency differed across time and context. Birds were consistent within seasons in both novelty responses, but they were only consistent between seasons in their responses towards novel people. Therefore, there may be greater constraints on individual plasticity towards stimuli that may resemble predators. Comparing behaviours that do and do not remain consistent offers insight into the costs and benefits of consistency (Carter, Goldizen et al., 2012, Dall et al., 2004). The lack of consistency in object neophobia cannot be explained by fluctuations in motivation because birds' food motivation ranks remained stable between seasons. Instead perhaps, object neophobia levels may respond much more to the social and environmental context than is often suggested in the nonhuman personality literature for traits such as neophobia or other forms of risk taking (e.g. Bell, Hankison, & Laskowski, 2009). Evidence is accumulating that temporary changes in the environment, such as changes in the recent or current social context, can influence the levels of otherwise stable risk taking (Jolles et al., 2016, Jolles et al., 2014) and the characteristics of individuals that innovate (Duffield, Wilson, & Thornton, 2015). Therefore interindividual differences in behaviour are not always maintained over time (Carter, Goldizen et al., 2012, Kluen and Brommer, 2013). This would not be the first time that individual consistency in neophobia has been found only during short time periods, and its variability has been deemed a result of individual differences in reaction norms across contexts (Kluen & Brommer, 2013). However, the fact that individual consistency varied in a different manner towards each of our types of novelty even when birds were tested in the same social group shows that individuals can show contrasting reaction norms for different types of avoidance behaviours. Further examination of the social and environmental influences on these norms and other types of personality traits will help to shape our understanding of the trade-offs and state-dependent nature of stable individual behaviours (Dall & Griffith, 2014).

Overall, the extent of seasonal change and inconsistency we observed in this study implies that caution should be taken when using neophobia as a sole measure of personality across time, and that variation in consistency across seasons should not be dismissed as noise. While it is not yet clear to what extent our results should be generalized to all social groups or species, the fact that we found such flexibility in our group suggests that neophobia is not always a stable trait. Critically, had either season been tested alone, we would have come to entirely different conclusions about the connections between neophobia, risk taking and dominance in this rook group. For example, during the breeding season individual approach ranks towards both types of novel stimuli were correlated, but outside the breeding season they were not. The seasonal difference in correlation between both stimuli types that we found may help explain why some studies have found links between types of neophobia (Bergvall et al., 2011, Verbeek et al., 1994) and others have not (e.g. Boogert et al., 2006, Fox et al., 2009). The existence of such variability opens up the exciting possibility that the direction and magnitude of seasonal change in risk taking may differ depending on the seasonal pressures and social structure that species experience. Glossing over such potential variability risks miscategorizing traits, and masking the drivers and consequences of novelty avoidance. Instead, neophobia might be better studied with the expectation that individuals will differ in how consistent they are across situations (e.g. Stamps, Briffa, & Biro, 2012). Therefore, although researchers may be able to assess meaningful variation via a neophobia test at a single time point, over longer periods, such a measure may not reflect maintained differences between individuals. Future studies will be essential to determining these dynamics, especially those that can replicate seasonal change across multiple social groups or differentiate it between species.

This study demonstrates that novelty responses can vary depending on the season, the type of novelty and the social consequences of novelty approach. Continued research into the mechanisms underlying such variation in neophobia is needed considering that novelty avoidance has ecological consequences and is often used in the personality literature. In particular, studies examining hormonal mechanisms and individual reaction norms could prove useful in disentangling individual variation from seasonal trends. However, it is critical that this variation is assessed in social settings for social animals, rather than on isolated individuals (Dall & Griffith, 2014). Such efforts will help determine when, where and why we expect to see stable individual differences in behaviour.

## Figures and Tables

**Table 1:** Survival models for latency to approach the food bowl

Variable	B	SE	*z*	*P*
Trial number	0.033	0.04	0.87	0.389
**Season (Breeding)**	**−0.211**	**0.29**	**−0.74**	**0.459**
**Aviary location (Table)**	**−0.484**	**0.13**	**−3.71**	**<0.001**
**Dominance**	**−0.110**	**0.03**	**−4.13**	**<0.001**
**Condition (Novel Object)**	**−0.658**	**0.30**	**−2.16**	**0.030**
Condition*Dominance	0.007	0.28	−0.24	0.810
Condition*Season	0.078	0.27	0.28	0.773
Season*Dominance	**0.102**	**0.03**	**3.42**	**<0.001**

Variable level is listed within parentheses. Statistically significant effects are in bold. The highest ranking bird was assigned a dominance of 1.

**Table 2:** Survival models for latency to approach experimenter

Variable	B	SE	*z*	*P*
Trial number	−0.007	0.03	−0.22	0.827
Aviary location (Table)	−0.148	0.12	−1.25	0.210
**Dominance**	**−0.058**	**0.02**	**−**2.82	**0.005**
**Condition (Novel Person)**	**−0.560**	**0.28**	**−1.96**	**0.050**
Season (Breeding)	0.518	0.27	1.928	0.054
Condition*Dominance	−0.014	0.03	−0.52	0.603
Condition*Season	**0.680**	**0.24**	**2.80**	**0.005**
Season*Dominance	−0.010	0.03	−0.39	0.700

Variable level is listed within parentheses. Statistically significant effects are in bold. The highest ranking bird was assigned a dominance of 1.

## References

[bib1] Altmann J. (1974). Observational study of behavior: Sampling methods. Behaviour.

[bib2] Apfelbeck B., Raess M. (2008). Behavioural and hormonal effects of social isolation and neophobia in a gregarious bird species, the European starling (*Sturnus vulgaris*). Hormones and Behavior.

[bib3] ASAB/ABS (2012). Guidelines for the treatment of animals in behavioural research and teaching. Animal Behaviour.

[bib4] Baugh A.T., van Oers K., Naguib M., Hau M. (2013). Initial reactivity and magnitude of the acute stress response associated with personality in wild great tits (*Parus major*). General and Comparative Endocrinology.

[bib5] Bebus S.E., Small T.W., Jones B.C., Elderbrock E.K., Schoech S.J. (2016). Associative learning is inversely related to reversal learning and varies with nestling corticosterone exposure. Animal Behaviour.

[bib6] Bell A.M., Hankison S.J., Laskowski K.L. (2009). The repeatability of behaviour: A meta-analysis. Animal Behaviour.

[bib7] Benson-Amram S., Holekamp K.E. (2012). Innovative problem solving by wild spotted hyenas. Proceedings of the Royal Society B: Biological Sciences.

[bib8] Bergvall U.A., Schäpers A., Kjellander P., Weiss A. (2011). Personality and foraging decisions in fallow deer, *Dama dama*. Animal Behaviour.

[bib9] Bókony V., Kulcsár A., Tóth Z., Liker A. (2012). Personality traits and behavioral syndromes in differently urbanized populations of house sparrows (*Passer domesticus*). PLoS One.

[bib10] Boogert N.J., Reader S.M., Laland K.N. (2006). The relation between social rank, neophobia and individual learning in starlings. Animal Behaviour.

[bib11] Brown G.E., Ferrari M.C.O., Elvidge C.K., Ramnarine I., Chivers D.P. (2013). Phenotypically plastic neophobia: A response to variable predation risk. Proceedings of the Royal Society B: Biological Sciences.

[bib12] Carter A.J., Goldizen A., Heinsohn R. (2012). Personality and plasticity: Temporal behavioural reaction norms in a lizard, the Namibian rock agama. Animal Behaviour.

[bib13] Carter A.J., Marshall H.H., Heinsohn R., Cowlishaw G. (2012). How not to measure boldness: Novel object and antipredator responses are not the same in wild baboons. Animal Behaviour.

[bib14] Chiarati E., Canestrari D., Vera R., Baglione V. (2012). Subordinates benefit from exploratory dominants: Response to novel food in cooperatively breeding carrion crows. Animal Behaviour.

[bib15] Cole E.F., Quinn J.L. (2014). Shy birds play it safe: Personality in captivity predicts risk responsiveness during reproduction in the wild. Biology Letters.

[bib16] Dall S.R.X., Griffith S.C. (2014). An empiricist guide to animal personality variation in ecology and evolution. Frontiers in Ecology and Evolution.

[bib17] Dall S.R.X., Houston A.I., McNamara J.M. (2004). The behavioural ecology of personality: Consistent individual differences from an adaptive perspective. Ecology Letters.

[bib18] Damsgard B., Dill L.M. (1998). Risk-taking behavior in weight-compensating coho salmon, *Oncorhynchus kisutch*. Behavioral Ecology.

[bib19] Davidson G.L., Clayton N.S., Thornton A. (2015). Wild jackdaws, *Corvus monedula*, recognize individual humans and may respond to gaze direction with defensive behaviour. Animal Behaviour.

[bib20] Dingemanse N.J., Kazem A.J.N., Reale D., Wright J. (2010). Behavioural reaction norms: Animal personality meets individual plasticity. Trends in Ecology and Evolution.

[bib21] Duffield C., Wilson A.J., Thornton A. (2015). Desperate prawns: Drivers of behavioural innovation vary across social contexts in rock pool crustaceans. PLoS One.

[bib22] Feare C.J., Dunnet G.M., Patterson I.J. (1974). Ecological studies of the rook (*Corvus frugilegus L.*) in North-East Scotland: Food intake and feeding behaviour. Journal of Applied Ecology.

[bib23] Ferrari M.C.O., McCormick M.I., Meekan M.G., Chivers D.P. (2015). Background level of risk and the survival of predator-naive prey: Can neophobia compensate for predator naivety in juvenile coral reef fishes?. Proceedings of the Royal Society B: Biological Sciences.

[bib24] Fournier D., Skuag H., Ancheta J., Ianelli J., Magnusson A., Maunder M. (2012). AD model builder: Using automatic differentiation for statistical inference of highly parameterized complex nonlinear models. Optimal Methods Software.

[bib25] Fox R.A., Ladage L.D., Roth T.C., Pravosudov V.V. (2009). Behavioral profile predicts dominance status in mountain chickadees, *Poecile gambeli*. Animal Behaviour.

[bib26] Frid A., Dill L. (2002). Human-caused disturbance stimuli as a form of predation risk. Conservation Ecology.

[bib27] Gamer M., Lemon J., Fellows I., Singh P. (2010). Package ‘irr’: Various coefficients of interrater reliability and agreement. https://cran.r-project.org/web/packages/irr/index.html.

[bib28] Green P.T. (1981). Some results from trapping rooks. Ringing & Migration.

[bib29] Greenberg R. (1989). Neophobia, aversion to open space, and ecological plasticity in song and swamp sparrows. Canadian Journal of Zoology.

[bib30] Greenberg R. (1990). Ecological plasticity, neophobia and resource use in birds. Studies in Avian Biology.

[bib31] Greenberg R. (1992). Differences in neophobia between naive song and swamp sparrows. Ethology.

[bib32] Greenberg R., Laland K.N., Reader S.M. (2003). The role of neophobia and neophilia in the development of innovative behaviour of birds. Animal innovation.

[bib33] Greenberg R., Mettke-Hofmann C. (2001). Ecological aspects of neophobia and neophilia in birds. Current Ornithology.

[bib34] Greggor A.L., Clayton N.S., Fulford A., Thornton A. (2016). Street smart: Faster approach towards litter in urban areas by highly neophobic corvids and less fearful birds. Animal Behaviour.

[bib35] Greggor A.L., McIvor G., Clayton N.S., Thornton A. (2016). Contagious risk taking: Social information and context influence wild jackdaws' responses to novelty and risk. Scientific Reports.

[bib36] Greggor A.L., Thornton A., Clayton N.S. (2015). Neophobia is not only avoidance: Improving neophobia tests by combining cognition and ecology. Current Opinion in Behavioral Sciences.

[bib37] Heinrich B., Marzluff J., Adams W. (1995). Fear and food recognition in naive common ravens. Auk.

[bib38] Jolles J.W., Aaron Taylor B., Manica A. (2016). Recent social conditions affect boldness repeatability in individual sticklebacks. Animal Behaviour.

[bib39] Jolles J.W., Boogert N.J., van den Bos R. (2015). Sex differences in risk- taking and associative learning in rats. Royal Society Open Science.

[bib40] Jolles J.W., Fleetwood-Wilson A., Nakayama S., Stumpe M.C., Johnstone R.A., Manica A. (2014). The role of previous social experience on risk-taking and leadership in three-spined sticklebacks. Behavioral Ecology.

[bib41] Jolles J.W., Ostojić L., Clayton N.S. (2013). Dominance, pair bonds and boldness determine social-foraging tactics in rooks, *Corvus frugilegus*. Animal Behaviour.

[bib42] Kluen E., Brommer J.E. (2013). Context-specific repeatability of personality traits in a wild bird: A reaction-norm perspective. Behavioral Ecology.

[bib43] Kotrschal K., Hirschenhauser K., Mostl E. (1998). The relationship between social stress and dominance is seasonal in greylag geese. Animal Behaviour.

[bib44] Lee W.Y., Lee S., Choe J.C., Jablonski P.G. (2011). Wild birds recognize individual humans: Experiments on magpies, *Pica pica*. Animal Cognition.

[bib45] Leiva D., Solanas A., Kenny D.A. (2010). DyaDA: An R package for dyadic data analysis. Proceedings of Measuring Behavior.

[bib46] Marzluff J.M., Walls J., Cornell H.N., Withey J.C., Craig D.P. (2010). Lasting recognition of threatening people by wild American crows. Animal Behaviour.

[bib47] Mettke-Hofmann C. (2000). Changes in exploration from courtship to the breeding state in red-rumped parrots (*Psephotus haematonotus*). Behavioural Processes.

[bib48] Mettke-Hofmann C. (2007). Object exploration of garden and sardinian warblers peaks in spring. Ethology.

[bib49] Miller R., Bugnyar T., Pölzl K., Schwab C. (2015). Differences in exploration behaviour in common ravens and carrion crows during development and across social context. Behavioral Ecology and Sociobiology.

[bib50] Nakagawa S., Schielzeth H. (2010). Repeatability for Gaussian and non-Gaussian data: A practical guide for biologists. Biological Reviews of the Cambridge Philosophical Society.

[bib51] Ouyang J.Q., Hau M., Bonier F. (2011). Within seasons and among years: When are corticosterone levels repeatable?. Hormones and Behavior.

[bib52] Patterson I.J., Dunnet G.M., Goodbody S.R. (1988). Body weight and juvenile mortality in rooks *Corvus frugilegus*. Journal of Animal Ecology.

[bib53] Pdulka S., Rohrbaugh R.W., Bonney R. (2004). Handbook of bird biology.

[bib54] Post P., Götmark F. (2006). Seasonal changes in Sparrowhawk Accipiter nisus predation: Prey vulnerability in relation to visibility in hunting habitats and prey behaviour. Ardea.

[bib55] R Core Team (2015). R: A language and environment for statistical computing. http://www.r-project.org/.

[bib56] Richard S., Wacrenier-Ceré N., Hazard D., Saint-Dizier H., Arnould C., Faure J.M. (2008). Behavioural and endocrine fear responses in Japanese quail upon presentation of a novel object in the home cage. Behavioural Processes.

[bib57] Romero L.M. (2002). Seasonal changes in plasma glucocorticoid concentrations in free-living vertebrates. General and Comparative Endocrinology.

[bib58] Shaffer J.P. (1995). Multiple hypothesis testing. Annual Review of Psychology.

[bib59] Shephard T.V., Lea S.E.G., Hempel de Ibarra N. (2014). ‘The thieving magpie’? No evidence for attraction to shiny objects. Animal Cognition.

[bib60] Stamps J.A., Briffa M., Biro P.A. (2012). Unpredictable animals: Individual differences in intraindividual variability (IIV). Animal Behaviour.

[bib61] Therneau T. (2015). A package for survival analysis in S. http://cran.r-project.org/package=survival.

[bib62] Thomas R., Bieber C., Arnold W., Millesi E. (2012). Living in a seasonal world: Thermoregulatory and metabolic adaptations.

[bib63] Vahl W.K., Lok T., Van Der Meer J., Piersma T., Weissing F.J. (2005). Spatial dumping of food and social dominance affect interference competition among ruddy turnstones. Behavioral Ecology.

[bib64] Verbeek M.E.M., Drent P.J., Wiepkema P.R. (1994). Consistent individual differences in early exploratory behavior of male great tits. Animal Behaviour.

[bib65] de Vries H. (1995). An improved test of linearity in dominance hierarchies containing unknown or tied relationships. Animal Behaviour.

[bib66] de Vries H. (1998). Finding a dominance order most consistent with a linear hierarchy: A new procedure and review. Animal Behaviour.

[bib67] Vrublevska J., Krama T., Rantala M.J., Mierauskas P., Freeberg T.M., Krams I.A. (2015). Personality and density affect nest defence and nest survival in the great tit. Acta Ethologica.

